# A Lumped Parameter Modelling Study of Idiopathic Intracranial Hypertension Suggests the CSF Formation Rate Varies with the Capillary Transmural Pressure

**DOI:** 10.3390/brainsci15050527

**Published:** 2025-05-20

**Authors:** Grant A. Bateman, Alexander R. Bateman

**Affiliations:** 1 Department of Medical Imaging, John Hunter Hospital, Newcastle, NSW 2310, Australia; 2 School of Medicine and Public Health, College of Health, Medicine and Wellbeing, Newcastle University, Callaghan Campus, Newcastle, NSW 2308, Australia; 3 School of Engineering, College of Engineering, Science and Environment, Newcastle University, Callaghan Campus, Newcastle, NSW 2308, Australia; alex.bateman@newcastle.edu.au

**Keywords:** anaemia, blood–brain barrier, cerebral blood flow, CSF formation rate, idiopathic intracranial hypertension, retinoic acid, tetracycline

## Abstract

**Background**: Idiopathic intracranial hypertension (IIH) is, by definition, of unknown cause. Davson’s equation indicates that the increased intracranial pressure (ICP) found in IIH could be due to an increase in the CSF formation rate (CSF_fr_), the CSF outflow resistance (R_out_) or the venous sinus pressure. Studies simultaneously measuring the ICP and sagittal sinus pressures in IIH suggest that there is either a reduction in the R_out_ and/or the CSF_fr_. The latter suggests that the increased venous pressure can be the only variable causing this disease process. A study maintaining the ICP at zero showed a significantly elevated CSF_fr_ in this disease. The purpose of the current study is to define the most feasible explanation for these findings and to suggest a viable pathophysiology for IIH. **Methods**: A lumped parameter vascular model, originally developed to study normal pressure hydrocephalus, was extended to investigate IIH. The model used the simultaneously obtained ICP and sagittal sinus pressure measurements from five experiments published in the literature to estimate the CSF_fr_ and the capillary transmural pressure (TMP). The assumptions made during this study were those of a normal mean arterial pressure, a normal total R_out_ and a normal blood flow rate. **Results**: When the CSF formation rates were plotted against the estimated capillary transmural pressures, a straight line was returned, suggesting that the CSF_fr_ and capillary TMP are related. **Conclusions**: The novel findings of this study suggest that the CSF_fr_ in IIH varies with the capillary TMP. A reduced capillary TMP in IIH can moderate the ICP if there is net CSF absorption across the capillaries. This would require the blood–brain barrier (BBB) to be disrupted. The model suggests that drugs which stabilise the BBB may trigger IIH by blocking CSF absorption across the capillaries, increasing the apparent CSF formation rate back toward normal and increasing the ICP. Anaemia will promote IIH by increasing the cerebral blood flow, the capillary TMP and the CSF_fr_.

## 1. Introduction

The clinical syndrome of idiopathic intracranial hypertension (IIH), also known as pseudotumor cerebri, occurs in patients who present with high pressure-type headaches and/or papilledema and visual obscuration. At lumbar puncture, the cerebrospinal fluid (CSF) pressures are elevated but the CSF composition is normal [1]. The diagnosis is made by lumbar puncture, with the accepted cutoff for the diagnosis of IIH being a CSF pressure elevation above 25 cm H_2_O in adults [2]. Currently, the disease is listed as idiopathic, meaning that the clause is not defined. Using Davson’s Equation (2), it has been argued that the elevated intracranial pressure (ICP) could come about by an increase in the CSF formation rate (CSF_fr_), an increase in CSF outflow resistance (R_out_) or an increased venous sinus pressure [3]. The pressure gradient between the ICP and the sagittal sinus depends on the CSF_fr_ multiplied by the R_out_. It has been suggested that, on the basis of a reduced pressure gradient between the CSF and sagittal sinus in IIH, the venous pressure, rather than an increase in the other two variables, is the underlying cause of IIH [4]. The cause of the reduced pressure gradient between the ICP and venous sinuses has not been previously investigated. There are three recent studies where the pressure gradient between the CSF and venous sinuses were measured at the same time and found to be 2.7 mmHg [5], 1.8 mmHg [6] and 1.1 mmHg [7] compared to a normal value of 4 mmHg [8,9]. Davson’s equation would suggest these results indicate either a significant reduction in the CSF_fr_ and/or the R_out_. Indeed, in one of the studies under consideration [6], the CSF formation rate was assumed to be normal by the authors and the outflow resistance was calculated to be 5.2 mmHg/mL/min, compared to a normal figure of approximately 10 mmHg/mL/min. Paradoxically, either a reduced formation rate or outflow resistance are the reverse of what would be expected to induce a raised CSF pressure. When mock CSF was infused into IIH patients by Lalou et al. [6], the ICP to sinus pressure gradient increased above normal to 4.9 mmHg, suggesting that either the outflow resistance or the CSF formation rate had changed during the infusion study. In a study where the ICP was maintained at 0 mmHg using a liquoGuard7 pump, the CSF formation rate in IIH was found to be 86 mL/h or 1.43 mL/min [10], compared to the normal value of 0.40 mL/min [6]. This later study effectively excluded the outflow resistance from consideration and, therefore, suggests that the CSF_fr_ is increased by over 250% in IIH. This indicates that the CSF_fr_ is likely to be the variable of interest in IIH, rather than the outflow resistance.

This raises an apparent paradox. The CSF_fr_ is apparently normal, increased or decreased in IIH depending on the circumstances. A hypothesis is tendered that the CSF_fr_ varies depending on the pressure gradient across the capillary walls within the brain. The purpose of the current study is to extend a lumped parameter vascular modelling study, initially performed to look at blood flow and pressure in normal pressure hydrocephalus [11], to incorporate the CSF formation rate and capillary transmural pressure (TMP), to test whether this is the most feasible explanation for this apparent paradox. Therefore, we wish to suggest a viable pathophysiology for this disease.

## 2. Materials and Methods

### 2.1. Equations

The study begins with Davson’s equation, which relates the intracranial pressure (ICP) to the CSF formation rate, the CSF outflow resistance and the venous sinus pressure [12]:

(1) $$ICP={CSF}_{fr}\times R_{out}+P_{sss}$$

where *ICP* is the intracranial pressure, *CSF_fr_* is the CSF formation rate, *R_out_* is the CSF outflow resistance and *P_sss_* is the pressure in the superior sagittal sinus. Next, Ohms law for hydraulic circuits is required:

(2) ∆P=Q×R

where *ΔP* is the pressure drop across a vascular segment, *Q* is the flow rate through the segment and *R* is the resistance. As resistances in series are directly additive, the following can be derived:

(3) $$R_{art}+R_{cap}+R_{ven}+R_{cuf}=R_{tot}$$

where *R_art_* is the arterial segment resistance, *R_cap_* is the resistance of the capillaries, *R_ven_* is the venous resistance, *R_cuf_* is the resistance of the venous outflow cuff and *R_tot_* is the total resistance for the entire vascular system. Poiseuille’s equation calculates the pressure drop across each of these segments:

(4) ΔP=8μLQπr4

where *ΔP* is the pressure drop, *µ* is the viscosity, *L* is the vessel length, *Q* is the fluid flow rate, *π* is the circle proportionality constant and *r* is the radius. Substituting Equation (2) into (4) and eliminating *Q* from both sides gives an equation for the resistance in each segment:

(5) R=8μLπr4

In this modelling study, the viscosity, the length of each vessel segment and *π* are constants, so it can be shown that a change in resistance for any segment depends only on a change in the vessel radius, i.e.,

(6) $$∆R=∆r^{-4}$$

The volume of a vessel is given by the equation for a cylinder, i.e.,

(7) $$V=L\pi r^{2}$$

where *V* is the volume, *L* is the vessel length and *r* is the radius of the vessel. Given that *L* and *π* are constants for any given segment, the change in volume is dependent on the change in radius, i.e.,

(8) $$∆V=∆r^{2}$$

Substituting Equation (8) into Equation (6) gives

(9) $$∆R=∆V^{-2}$$

The next equation relates the transmural pressure across a vessel to the vessel cross-sectional area [13]:

(10) $$P_{tm}=\frac{4Eh}{{3R}_{o}}(1-\sqrt{\frac{A_{o}}{A}})$$

where *P_tm_* is the transmural pressure across the vessel wall (lumen pressure- CSF pressure), *E* is the circumferential Young’s modulus of the vessel wall, *h* is the wall thickness, *R_o_* is the radius in the stress-free state, *A_o_* is the area in the stress-free state and *A* is the area following the applied transmural pressure. This equation was previously used to show that the volume of the venous outflow varies with the transmural pressure by the Equation [11]:

(11) $${∆TMP}_{ven\;\;}=-0.033{{\Delta V}_{ven}}^{2}+7.49\times {\Delta V}_{ven}-3.44$$

where *ΔTMP_ven_* is the normalised increase in venous transmural pressure and *ΔV_ven_* is the change in venous volume.

### 2.2. Model Input Parameters

The input parameters are unchanged from the previous study [11] and will only be briefly described as the details can be obtained from the original study. This study is based on a middle-aged individual with a brain size of 1500 g. A normal global CBF is 50 mL/100 g/min [11], giving a normal cerebral blood arterial inflow of 750 mL/min. The normal mean arterial inflow pressure is 100 mmHg [14]. The normal precapillary bed pressure is 32 mmHg [15]. The end capillary pressure is estimated to be 15 mmHg [16]. The normal CSF pressure in middle age is 11.5 mmHg [17] and the normal pressure gradient from the CSF to the superior sinus lumen is 4 mmHg [8,9], giving a normal sinus pressure by subtraction of 7.5 mmHg [18]. The normal transmural pressure of the subarachnoid cortical veins in primates is 2.5 mmHg [19]. Using this figure for the model, it can be seen that the pre-venous outflow cuff pressure is 14 mmHg by addition of the TMP to the ICP.

In a 1500 g brain, the total CBV would be 51 mL [11]. Hua et al. found the arterial component of the CBV to be 25% [20] or 12.8 mL in total. This leaves the remaining 75% for the capacitance vessels, including the veins and capillaries or 38.2 mL. The estimated percentage of this latter figure for the capillaries is 53% [21], giving a total capillary blood volume of 20.3 mL and a total venous blood volume of 17.9 mL.

The normal CSF outflow resistance (R_out_) can be calculated using Equation (1). Given this, the normal CSF_fr_ is 0.4 mL/min [22] and the normal pressure gradient between the ICP and sinus pressure is 4 mmHg [8,9], giving a resistance of 10 mmHg/mL/min.

### 2.3. Vessel Responses to Transmural Pressure Variations

It is assumed that variations in the arterial resistance and volume in this model depend entirely on the arterial autoregulation and muscle tone and not the vessel transmural pressure. As the arterial pressure is always much higher than the ICP, the arterial transmural pressure will have no effect on the outcome of the current modelling study.

In the capillary bed, the vessels do not actively alter their diameter [23], indicating that they react purely to their transmural pressure. In a rat model, extreme hyperventilation decreased the PCO_2_ from 40 to 21.6 mmHg without affecting PO_2_; the capillary size was not significantly different to controls, despite the expected arteriolar constriction [24]. However, in the opposite case, in rats made extremely hypercapnic secondary to hypoventilation, the PCO_2_ increased to 95.6 mmHg but PO_2_ was normal; the capillary diameter increased by 20%, consistent with a 44% increase in volume compared to known control values [24]. Thus, a moderate reduction in capillary TMP does not change the capillary size but a maximal increase in TMP increases their volume by 44%. To simplify the current study, it is assumed that the volume of the capillaries vary, between normal and maximally dilated, as a linear function of their transmural pressure. A previous study indicated that an increase in capillary TMP from 12 to 37.9 mmHg would increase the capillary volume by 44% or a 1.7% increase in volume for each 1 mmHg pressure rise. Below a TMP of 12 mmHg, the volume is unchanged at 20.3 mL and above a TMP of 37.9, the elastic limit is reached and the volume is set to 29.2 mL.

Similarly to the capillaries, the veins alter their size purely depending on their transmural pressures. In a previous modelling study [11], the function for the outflow vein dilatation was found to be summarised by Equation (11).

At the distal end of the cortical veins, as they join the sinus wall, the outflow cuff segment resides. The collapse of this segment occurs physiologically and is passively modulated by the transmural pressure between the ICP and the sinus pressure, which is usually negative [25]. The segment is very short, and as it is mostly under a state of collapse with physiological ICPs, the change in volume from this segment will be ignored in this model. This is despite acknowledging that the cuff is dilated in the drainage model. However, its resistance will be taken into consideration. In the previous study four, differing cuff transmural pressures resulted in four differing resistances [11]. When these points were plotted, a line with an R^2^ of 0.998 resulted, suggesting that the cuff resistance varies as a linear function of the cuff TMP, thus giving Equation (12):

(12) $$R_{cuf}=-2.71\times {TMP}_{cuf}+0.008$$

where *R_cuf_* is the cuff resistance and *TMP_cuf_* is the cuff transmural pressure.

The sagittal sinus pressure will be varied as per the literature being modelled.

## 3. Results

### Idiopathic Intracranial Hypertension

The two input variables and the main modelling findings are summarised in Table 1.

**Figure 1:**
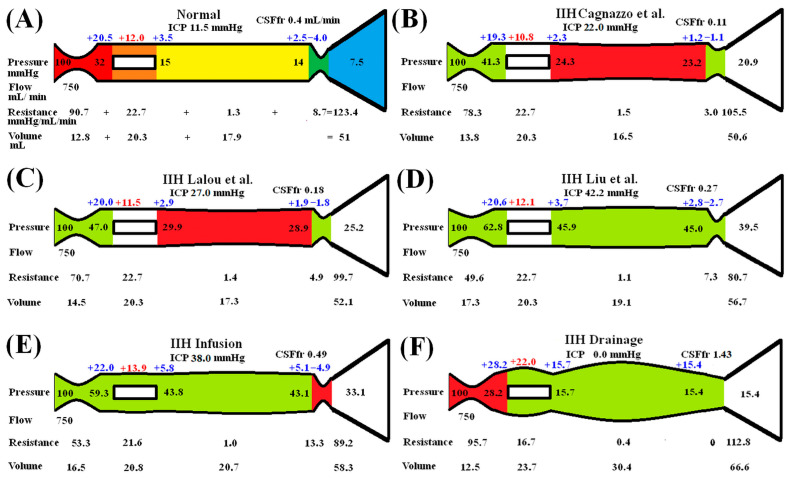
Results of modelling. (**A**) Normal findings. The red segment is the arterial, orange is the capillary, yellow is the veins, green is the outflow cuff and blue is the venous sinus. The vascular pressures are shown within the vessels. The blue numbers are the transmural pressures at each site. The red number is the average capillary transmural pressure. The resistances and volumes for each segment are shown below the vessel. The calculated CSF formation rate has been appended. (**B**) The findings in IIH from the study by Cagnazzo et al. [7]. The red area indicates an increase in resistance in the veins and the green decreased resistance compared to normal. Both the capillary TMP and CSF_fr_ are reduced compared to normal. (**C**) The findings in IIH from the study by Lalou et al. [6]. Both the capillary TMP and CSF_fr_ are increased compared to (**B**). (**D**) The findings in IIH from the study by Liu et al. [5]. Both the capillary TMP and CSF_fr_ are increased compared to (**B**). (**E**) The findings in IIH from Lalou et al. [6]. following an infusion study. The capillary TMP and CSF_fr_ have increased compared to the baseline study seen in (**C**). (**F**) The findings in IIH following CSF drainage, as found by Tariq et al. [10]. Both the capillary TMP and CSF_fr_ have significantly increased. Figure 1A. Reproduced from [11] under a CC BY 4.0 commons licence.

Note that there is a progressive increase in the CSF_fr_ for each study as you pass down the list. This is associated with an increase in capillary TMP. An expanded presentation of the modelling results is provided in Figure 1. The five vascular segments modelled are shown in Figure 1A, with the arterial segment shown in red, the capillaries in orange, the veins in yellow, the outflow cuff in green and the sinus in blue. The pressures obtained from the literature have been appended to the beginning and end of each vascular segment within the vessels in Figure 1A. Given the arterial inflow volume passes through each segment sequentially, the resistance of each segment can be calculated using Equation (2). These resistances are appended below the vessels in Figure 1. The normal cerebral blood volume (CBV) values for each segment and the total CBV have been obtained from the literature and are shown below the resistances. The blue numbers represent the transmural pressure gradients between the pressure at the beginning and end of each capacitance vessel segment and the ICP, and are obtained by subtraction. The red figure is the average capillary TMP obtained by averaging the TMP before and after the capillaries. The CSF_fr_ has been appended in the top right. Figure 1B–F represent the effects of the differing alterations in the perfusion pressure from the differing studies being modelled. In these figures, the red segments represent the areas of increased resistance compared to the normal findings and the green represent reduced resistance.

In Figure 1B, the findings from the study by Cagnazzo et al. [7] have been modelled. The ICP is 22 mmHg and the sinus pressure 20.9 mmHg. The arterial inflow has been kept at the normal level in keeping with the majority of studies, indicating that the flow rate is normal in IIH [26]. The pressure drop across the entire system is known from the data as supplied and the total blood flow is specified, so the total resistance is calculated using Equation (2). The TMP across the outflow cuff is obtained by subtracting the ICP from the sinus pressure and is reduced below normal. The cuff outflow resistance can be calculated from Equation (12) and is also reduced. Knowing the cuff resistance and blood flow will set the blood pressure at the end of the veins by using Equation (2). The reduction in TMP across the veins reduces their volume using Equation (11). The change in venous resistance can be calculated using the change in venous volume using Equation (9). This allows the post capillary pressure to be calculated using Equation (2). Using the capillary tube law as defined, the volume and resistance of this segment will be unchanged from normal. Knowing the total resistance and the other resistances, the arterial inflow resistance can be calculated using Equation (3). Note that there is a reduction in both the arterial and the outflow cuff resistance with a reduction in resistance overall compared to normal. The calculated average capillary TMP is reduced by 10% compared to the normal value. The CSF_fr_ is estimated to be 0.11 mL/min using Davson’s equation, or 73% less than normal.

In Figure 1C, the baseline findings found in the study by Lalou et al. [6] (i.e., an ICP of 27 mmHg and superior sagittal sinus pressure of 25.2 mmHg) have been modelled. The calculated arterial resistance is less than in Figure 1B but the cuff resistance is increased. The capillary TMP is reduced by 4% compared to normal. The calculated CSF_fr_ is 0.18 mL/min, or 55% less than normal.

In Figure 1D, the study by Liu et al. is modelled. The baseline ICP and sagittal sinus pressures were 42.2 mmHg and 39.5 mmHg in IIH, respectively. The calculated capillary TMP from this study is normal at 12.1 mmHg, and the CSF_fr_ is 0.27 mL/min, or 33% less than normal.

Figure 1E models the findings in the study by Lalou et al. following their infusion of mock CSF into the subarachnoid space in IIH [6]. The ICP was found to be 38 mmHg and the sinus pressure 33.1 mmHg. Using the same technique as for Figure 1B, the total resistance is reduced compared to the pre infusion study results in Figure 1C (despite the cuff resistance being increased), due to the effect of the arterial resistance dropping. The capillary TMP has increased by 16% compared to normal, and the CSF_fr_ has increased by 72% compared to the pre infusion study.

Figure 1F models the effect of draining the CSF using the LiquoGuard7 device, as used in the study by Tariq et al. [10]. The effect of dropping the ICP to zero is to effectively treat the IIH. In IIH patients, the venous transverse sinuses show narrowing (a stenosis), which often rapidly returns to normal following a lumbar puncture [27]. This is the equivalent to the dilatation of the transverse sinuses, which occurs in successful stenting procedures in IIH, i.e., the pressure gradient along the transverse sinuses returns to normal. The superior sagittal sinus pressure following a successful sinus stenosis stent remains higher than normal at 15.4 mmHg [28]. This appears to be due to the stent failing to treat the raised venous pressure secondary to the obesity induced central venous pressure elevation. Therefore, an outflow pressure of 15.4 mmHg has been modelled in Figure 1F. The model indicates that the outflow cuff is maximally dilated, and the resistance of this short segment is reduced to zero. The vastly increased venous TMP maximally dilates the veins by 70% and reduces their resistance. The capillary TMP is increased and the capillary volume will increase. The capillary volume and resistance have been adjusted to allow for this effect. The overall result is to increase the capillary TMP by 83% above normal. The CSF_fr_ was measured directly by Tariq et al. and found to be elevated at 1.43 mL/min [10], or at an increase of 258% above normal.

## 4. Discussion

As discussed in the introduction, there seems to be some variability in either the CSF outflow resistance (R_out_) or the CSF formation rate (CSF_fr_) in IIH. Lalou et al. [6] directly measured the background ICP and venous sinus pressure in IIH, finding mean values of 27.0 mmHg and 25.2 mmHg, respectively. They assumed a normal CSF_fr_ and using Davson’s Equation (2), obtained a R_out_ of 5.2 mmHg/mL/min. This is 48% less than the normal figure of 10 mmHg/mL/min. Using a similar technique, Liu et al. [5] found an average ICP in IIH of 42.2 mmHg and sinus pressure of 39.5 mmHg, which would give a R_out_ of 6.7 mmHg/mL/min, or 33% less than normal. Cagnazzo et al.’s figures [7] would give a R_out_ of 2.8 mmHg/mL/min, or 73% less than normal. Finally, Lalou et al. [6] performed an infusion test on their cohort [6]. According to their methodology, the average rate of mock CSF infusion was 1 mL/min, giving an apparent total CSF formation rate of 1.40 mL/min. During the infusion, the average ICP was 38 mmHg and the SSS pressure 33.1 mmHg. Placing these data into Equation (2) gives a R_out_ of 3.5 mmHg/mL/min, or 65% below normal. It is difficult to conceive of a mechanism whereby a very low resistance of 5.2 mmHg/mL/min before the infusion could fall even further to 3.5 mmHg/mL/min over the space of 10 min during the infusion study and then bounce back to normal when finished. If, rather than the CSF formation rate being a constant, the R_out_ was made a constant, then the CSF formation rate would vary. If the underlying R_out_ were normal, then the studies as discussed would give CSF formation rates of 0.11 mL/min, 0.18 mL/min and 0.27 mL/min and the post-infusion study data would yield 0.49 mL/min, respectively. As already discussed, in the study by Tariq et al. [10], the CSF was drained to maintain an ICP of 0 mmHg. The effect of this is to place an additional CSF outflow resistance (with an effective resistance of zero) in the system in parallel to all the others. Thus, all of the other normal outflow resistances are effectively excluded from the measurement. The CSF_fr_ was found to be 1.43 mL/min in IIH [10], or an increase of over 250%. Given the large change in CSF_fr_ in the final study, it would seem more likely that the CSF_fr_ is varying in IIH rather than the R_out_. This assertion is backed up by a study by Welch and Friedman, who directly measured the resistance through the arachnoid granulations. In this experiment, the outflow resistance of the arachnoid granulations varied with the pressure gradient across their walls [29]. In a recent study, the arachnoid granulations have been found to be smaller in IIH having a median cross-section of 5 mm^2^ compared to controls at 7 mm^2^ [30]. The authors noted that the smaller granulations should lead to reduced CSF absorption from a greater outflow resistance. Welch and Friedman directly measured the resistance to flow across the sampled arachnoid granulations and found this to be true [29]. They found that the granulations consisted of a labyrinth of small tubules of diameter 4–12 µm. The tubes were open to both the subarachnoid space and the venous sinus lumen. If the pressure gradient from the CSF to the sinus was reduced, the granulations reduced in size and the tubules within them were no longer discernible, suggesting a valve-like action. When the granulations were perfused with mock CSF, the critical opening pressure for the tubules was 1.5–3.7 mmHg. This provides a reason why the CSF pressure always sits approximately 4 mmHg above the venous sinus pressure, i.e., this is the pressure gradient set within the valves of the arachnoid granulations before they open. Note that the lowest pressure gradient, as found in the study by Cagnazzo et al. [7], would also have the lowest theorised R_out_ if the CSF_fr_ were a constant. If a low-pressure gradient pressure reduces the granulation size by underfilling them, then they should have a large actual resistance and not a decreased one. Thus, the CSF_fr_ is likely to be varying and not the total R_out_. Therefore, the purpose of the current study is to model the blood flow and pressure of the intracranial system in IIH, to try to find a feasible solution to account for these findings.

### 4.1. Variation in CSF_fr_ with ICP

**Figure 2:**
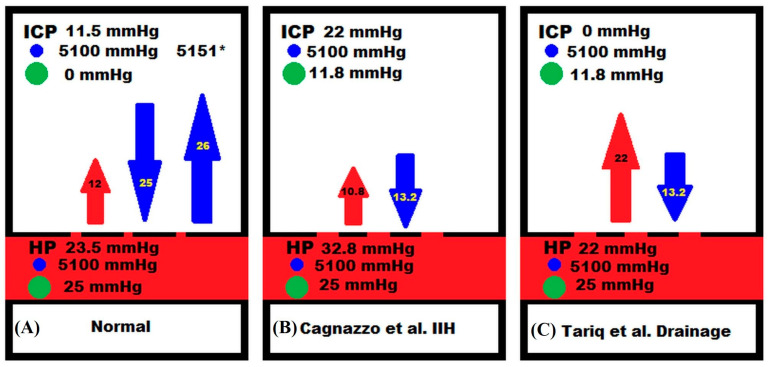
Correlation between capillary TMP and fluid flow. (**A**) The normal findings. The red segment is the capillary, the white the interstitial space. The small gaps in the upper wall of the capillary represent the aquaporin-4 water channels. These allow free passage of water but block salt and protein. ICP is the intracranial pressure. HP is the capillary hydrostatic pressure. The blue dot represents the salt and its osmotic pressure is beside it. The green dot represents the protein with its osmotic pressure is beside it. The red arrow represents the capillary TMP which is the ICP-HP. The middle blue arrow is the difference between the salt and protein osmotic pressure between the interstitium and the capillary. Subtracting the blue arrow from the red reveals the net pressure. This would tend to force water into the capillary at −13 mmHg. Despite this, no fluid can actually flow. Water absorption causing a 1% change in the interstitial volume would increase the interstitial osmotic pressure by 51 mmHg to 5151 as denoted with the asterisk, reversing the net osmotic pressure (right blue arrow) and reversing the fluid flow. (**B**) The findings in IIH from the study by Cagnazzo et al. [7]. The defects in the capillary wall are now larger, reflecting BBB breakdown. There is free flow of water and also salt, but the wall is semipermeable to protein. The capillary TMP is smaller than in the normal study. The movement of proteins has left a net osmotic gradient of 13.2 mmHg, as predicted by Equation (14). There is net fluid absorption into the capillary due to a net pressure gradient of −2.4 mmHg. (**C**) The findings in the study by Tariq et al. [10]. The capillary TMP has increased but the osmotic gradient is the same as (**B**). The net pressure gradient is +8.8 mmHg, favouring increased CSF production. *: This denotes the increased osmotic pressure, as described.

It is generally thought that the CSF_fr_ does not change with the ICP and remains a constant [31]. CSF is produced from differing regions within the brain. In total, 70% of CSF production comes from the choroid plexus, 18% from the capillaries from active transport and 12% from glucose metabolism [32]. This represents a relatively fixed CSF production. A CSF production less than 0.4 mL/min over the short term would imply that there is net absorption occurring somewhere other than the arachnoid granulations and a production greater than 0.4 mL/min implies an increase in production from somewhere other than the choroid plexus. The only possible instantaneously variable component of the CSF production could come from the capillaries, but this is excluded if the blood–brain barrier (BBB) is intact. This will be explained with the help of Figure 2.

**Figure 3:**
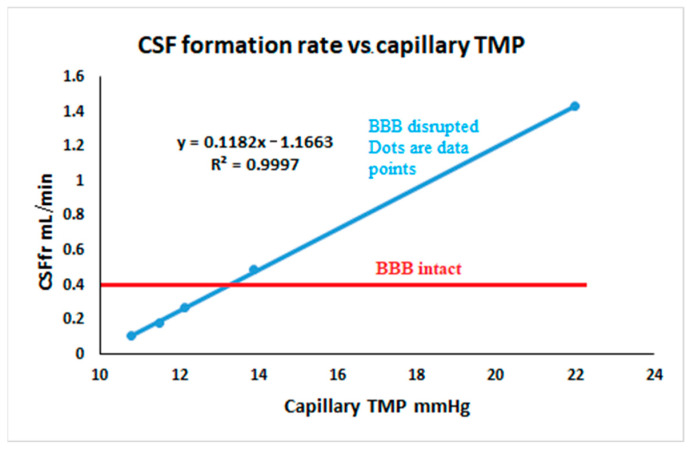
CSF formation rate vs. capillary transmural pressure.

Net capillary CSF production or absorption is expected to follow the Starling forces relationship. This is modelled using the following equation:

(13) $$J_{cap}=L_{cap}[\left\{P_{cap}-P_{CSF}\right\}-\sigma_{cap}\left\{\pi_{cap}-\pi_{CSF}\right\}]$$

where *J_cap_* is the net capillary fluid flow rate, *L_cap_* is the capillary hydraulic conductivity coefficient, *P_cap_−P_csf_* is the hydraulic pressure gradient across the capillary wall (i.e., the TMP), *σ_cap_* is the osmotic reflection coefficient and *π_cap_−π_csf_* is the osmotic pressure gradient across the capillary wall taking into account both salt and protein [33]. Note that at steady state, there is no significant difference in hydrostatic pressure between the CSF and the brain parenchyma within the limits of the accuracy of the equipment used to make these measurements [34,35]. With an intact BBB, the cerebral capillaries have hydraulic conductivities that are two to three orders of magnitude less than systemic capillaries (effectively zero) [36]. The osmotic reflection coefficient is 1 for all substances [33]. In Figure 2A, the capillary is shown with breaks in its wall, consistent with aquaporin-4 channels. These readily allow water to pass but do not allow salt or protein to pass [37]. The salt-derived osmotic pressure gradient across the capillary wall is close to zero because the osmotic pressures, although high at 5100 mmHg, match each other [33] (Figure 2A). The oncotic gradient due to protein is 25 mmHg higher in the capillaries than the interstitial fluid [38]. Note that the net pressure across the capillaries is −13 mmHg, which would tend to absorb water. However, this does not occur due to the BBB. Any absorption of water by the capillary occurring secondary to the net pressure, with an intact BBB, would rapidly increase the osmotic pressure difference between the plasma and the interstitium. This is because the salt does not follow the water due to the high osmotic reflection coefficient. Thus, as shown to right of Figure 2A, absorption of water from the interstitium leading to a 1% change in volume would increase the salt osmotic pressure by 51 mmHg, leading to an overall increase in hydrostatic and osmotic pressure of +38 mmHg. This effectively reverses the original flow of fluid [33]. However, this feedback control of water flow into the brain fails as the BBB breaks down and the net result is a change in brain water and the ICP [33]. This is because the hydraulic conductivity would increase and the osmotic reflection coefficient would decrease. In IIH, there is evidence of a significant disruption of the BBB. The capillaries of patients with IIH show vacuolization and degeneration of the basement membrane with degeneration of the pericyte processes. There is marked evidence of BBB leakage with the passage of blood borne fibrinogen and fibrin. None of these changes were seen in the controls [39]. The barrier breakdown probably occurs secondary to the elevated venous pressure because there is also BBB disruption in a mouse model of raised cerebral venous pressure [40]. This compares to normal pressure hydrocephalus where a BBB breakdown occurs despite a normal sinus pressure [11]. In Figure 2B, the findings from Cagnazzo et al. [7] have been illustrated. The gaps in the capillary wall are now larger to suggest that the salt can freely move. The membrane is semi-permeable to protein, depicted as a pore size where the protein can partly move through the wall by deforming because the pore size is just smaller than the protein. If the osmotic reflection coefficient is low for salt, then the salt will follow the water, and the capillary salt-derived osmotic pressure gradient will not increase. From the Starling forces Equation (13), it can be predicted that the capillary fluid flow (i.e., the J_cap_) will be a linear function of the capillary TMP, with the hydraulic conductivity being the slope of the line. Figure 3 is a plot of the five experiments from the literature, where we estimated the CSF_fr_ (from their data by using a normal R_out_) vs. our estimates for the capillary TMP from the modelling (see Figure 1 for the TMP and CSF_fr_ results). Note that this graph returns a blue line with an R^2^ of 0.99. The degree of correlation adds weight to the suggestion that the CSF_fr_ varies with the capillary TMP in IIH. The likelihood that the results of the four differing authors, as modelled, could so closely align by chance alone is very small. To illustration this fact, the results of a study into moderate Alzheimer’s disease (where the BBB is also disrupted), performed using the same model, gave a capillary TMP of 8.9 mmHg and CSF_fr_ of 0.18 mL/min [41]. A study into normal pressure hydrocephalus using the LiquoGuard7 device by Tariq et al. gave a CSF_fr_ of 1.32 mL/min [10] and our modelling gave a capillary TMP of 15.5 mmHg [42]. Both of these points are a long way to the left of the blue line in Figure 3. The red line in this figure is the expected constant CSF_fr_ if the BBB were intact.

A graph of the five calculated CSF formation rates vs. the transmural pressures for the studies obtained from the literature in blue. The red line is the expected unchanged CSF formation rate if the blood–brain barrier were intact, i.e., 0.4 mL/min. Note that the three baseline studies show a CSF formation rate which is below normal, suggesting that capillary absorption must be occurring if there is a fixed 0.4 mL/min CSF formation rate from the other sites.

A review of the equation for the blue line in Figure 3 suggests that the slope of the line, i.e., 0.12 mL/min/mmHg (as already discussed), equates to the hydraulic conductivity coefficient in Equation (13) because “x” in the equation represents the difference in hydrostatic pressure between the capillaries and interstitium. This figure would be 0.008 mL/min/mmHg/100 g of brain tissue for a 1500 g brain. This is 36% lower than the figure for skeletal muscle, which is 0.01–0.015 mL/min/mmHg/100 g [43] but obviously much higher than the normal figure for the brain capillaries, which is effectively zero. The surprising finding in Figure 3 is that the three baseline IIH studies show a significant reduction in the CSF_fr_ below normal, despite either a normal or only mildly reduced transmural capillary pressure. This suggests that something other than the hydrostatic pressure is decreasing the apparent CSF_fr_ once the BBB opens. A downregulation of the choroid plexus fluid formation could reduce the overall CSF production. A reduction in the choroid plexus CSF production has been previously suggested in the literature. A study with a mixed population of patients with elevated ICP from hydrocephalus and IIH showed that the CSF formation rate tended to decrease with the chronic elevation in ICP [44]. However, if this were the only factor reducing the flow, then the blue line in Figure 3 would be lower than the red line but still parallel to the red line, with no change in CSF_fr_ occurring with the variations in capillary TMP. Therefore, the difference in osmotic pressures between the capillaries and interstitial fluid must be the factor related to reducing the CSF_fr_. We can reproduce the equation for the blue line in Figure 3 to make it conform to the Starling forces Equation (13) to discover a cause for this effect. Note that we are only interested in the capillary component of the CSF formation rate, so we need to subtract the fixed CSF production rate of 0.4 mL/min originating from the choroid plexus production from each of the CSF_fr_ points in Figure 3.

(14) $$J_{cap}=0.12\left[\left\{P_{cap}-P_{CSF}\right\}-0.53\left\{25-0\right\}\right]$$

We can see that the final constant from the blue line equation is made up of the hydraulic conductivity coefficient, the reflection coefficient and the difference in the protein oncotic pressure between the capillary and the interstitial fluid (the salt shows no difference in osmotic effect). The estimated reflection coefficient is 0.53. If the fixed rate of CSF production from the choroid plexus and elsewhere were downregulated, then the reflection coefficient would be reduced. It becomes 0.39 if there is no fixed choroid/brain fluid production at all, but then the capillaries would need to supply all of the CSF production. These figures compare to the pulmonary capillary endothelium, which has a reflection coefficient to albumin of 0.5–0.64 [45]. The reflection coefficient measures the percentage of the difference in the osmotic pressure which is available to shift fluid [33]. A measure of 0 means that the osmolytes will equilibrate and, therefore, no gradient will remain to shift fluid and 1 indicates no passage of the osmolyte occurs and a full osmotic gradient is available. Thus, provided the reflection coefficient is zero for salt but remains semipermeable for protein, then the CSF formation rate will be lower than normal with an open BBB due to the absorption of fluid across the capillaries, provided the capillary transmural pressure stays below 13.1 mmHg. Above this level, the capillary TMP takes over to increase the overall fluid production. In Figure 2B, the semi-permeable nature of the capillary wall to proteins in IIH maintains a 13.2 mmHg osmotic gradient across the capillary wall. Subtracting this from the capillary TMP gives a net pressure gradient of −2.4 mmHg, meaning that there is net absorption of water across the capillary wall. Note that the model suggests that 0.49 mL/min is being absorbed across the arachnoid granulations in Figure 1E, despite mock CSF being infused at a rate of 1 mL/min during the infusion in this study. This indicates that at least 0.5 mL/min must be being absorbed by an accessory pathway, or a higher rate if choroid CSF production remains. Such a high flow across an accessory pathway would probably overwhelm the perineural and lymphatic systems. The perineural and lymphatic systems drain approximately 50% of the CSF production in sheep, with the remainder absorbed by the arachnoid granulations [46] but this amounts to only 0.03 mL/min in this species [47]. The human brain is much larger than that of a sheep and the CSF production is also much larger. It is suggested that CSF drainage from the nasal submucosal lymphatics may be a minor pathway in humans and primates due to differences in the relative sizes of the cribriform plates [48]. Thus, in IIH in humans, the capillaries are the most likely candidate for the increased accessory CSF absorption. In Figure 2C, the findings from Tariq et al. [10] are shown. The capillary TMP is larger than the residual protein osmotic gradient and there is a net +8.8 mmHg pressure gradient driving the increased CSF_fr_, as noted by capillary fluid transudation.

### 4.2. Clinical Utility and an Explanation of Pharmacological Effects

This modelling study suggests that IIH is a disease triggered by venous hypertension, which secondarily opens the blood–brain barrier and alters CSF production/absorption. The study predicts that maximum benefit will arise from reducing the venous pressure either by weight-loss, in the case of obese patients, or by venous sinus stenting in the case of transverse sinus stenosis. Other therapeutic treatments such as Acetozolamide medication, ventriculoperitoneal shunting or optic nerve fenestration may improve the symptoms but if the venous hypertension persists (due to ongoing obesity for example), then the BBB may remain impaired.

It is apparent that the CSF_fr_ is already low in IIH. Acetozolamide works by reducing the choroid CSF production further to reduce the ICP [49]. However, the already low inherent CSF_fr_ would blunt the effect of this drug. Also, reducing the ICP by this method will tend to increase the capillary TMP (similar to the findings in Figure 1F) and tend to increase the CSF_fr,_ partially reversing the effect. It is interesting to note that a Cochrane review concluded that there was insufficient evidence to recommend or reject the efficacy of Acetozolamide for the treatment of IIH [50]. Our findings suggest a reason for this low clinical utility.

Paradoxically, the opening of the blood–brain barrier may be partially beneficial in IIH. It will allow the ICP to be moderated by absorbing some CSF into the capillary bed if the TMP is low enough. This effect reduces the apparent total CSF formation rate to below normal (see Figure 2). The modelling would predict that the stabilisation of this blood–brain barrier disruption would increase the ICP by not allowing for some net absorption of CSF via the capillaries and increasing the CSF_fr_ back toward normal. There are two classes of drugs known to stabilise the blood–brain barrier: tetracycline-based antibiotics [51,52] and retinoic acid compounds [53]. Doxycycline reduces microvascular hyperpermeability and increases tight junction integrity [52]. Similarly, retinoic acid compounds improve the BBB by upregulating barrier related gene expression [53]. These two classes of compounds are both known to trigger IIH in those predisposed to the disease [54,55], suggesting that blood–brain barrier stabilisation is not an ideal strategy in IIH.

Glucocorticoids are known to have a beneficial effect in IIH in the short term [56] and are also known to stabilise the blood–brain barrier [57]. This finding would tend to refute the preceding paragraph; however, the effect of steroids is wide ranging. Steroids reduce the CSF formation rate in rabbits by 43% [58] and this effect may counter any BBB stabilising effect. It is also known that rapid steroid withdrawal will produce a rebound increase in ICP [56]. In dogs, rapid steroid withdrawal leads to a 68% increase in the CSF outflow resistance [59]. The latter would tend to suggest that the cause of the rebound is not purely related to the BBB.

### 4.3. Variations in Cerebral Blood Flow in IIH

As already discussed, the average cerebral blood flow in IIH has been found to be normal in IIH. However, the literature in this area varies considerably. It appears there are patients who have a lower than normal CBF associated with obesity (a reduction of about 12%) [60] and those with higher than normal CBF associated with anaemia (up to a 50% increase in CBF) [26]. Changing the CBF requires a change in the arterial resistance, all else being equal. In a previous modelling study of NPH, it was noted that reducing the CBF by 25% reduced the capillary TMP by 34% and increasing the CBF by 25% increased the TMP by 28%. Such a large effect occurs because there is a simultaneous change in the arterial, capillary and venous pressures and possibly the venous sinus outflow pressure as well. Therefore, the reduction in CBF noted to occur in obesity may be partly beneficial in IIH because it would tend to reduce the CSF formation rate and the ICP. This is despite an overall elevation in venous pressure increasing the CSF_fr_. Similarly, we can see that anaemia, which occurs in up to 20% of IIH patients [61], can promote IIH by increasing the capillary TMP and therefore the CSF formation rate in those who are predisposed to this disease.

### 4.4. Justification of Assumptions and Limitations

There are many assumptions inherent in the lumped parameter modelling. We can test the modelling we have performed by comparing the outcomes predicted by the model with the literature. Previously, the model successfully predicted the cerebral blood volumes changes in the literature found at the limits of autoregulation, in human and animal models [11], and the cerebral blood volume changes in Alzheimer’s disease [41]. In IIH, a large review indicated the mean initial ICP in this disease is 29.5 mmHg [62]. This figure is closest to the ICP found in Figure 1C of our modelling. The modelling suggests the total blood volume should not be significantly different, averaging 52.1 mL, compared to normal at 51 mL (2% different). Two studies have found a normal cerebral blood volume in IIH [63,64]. One study found a 33% increase in CBV in IIH [65]. However, the methodology in this paper was flawed. They measured the CBV with one technique (carbon monoxide red cell labelling) but compared the findings to controls using another method (fluorescence) [65]. Comparing their results to the normal red cell findings in an earlier paper by the same group [66] showed no significant difference in the CBV in IIH. Thus, our modelling appears to be accurate enough for the current purposes.

Poiseuille’s equation requires flow through a thin, rigid, circular tube of a Newtonian fluid, without turbulence. Extensive modelling has suggested that there is no significant difference between the results of the Newtonian and non-Newtonian assumptions in the intracranial blood vessels [67]. Trans-cranial Doppler data in the arterial tree of the brain [68] gives a calculated Reynolds number of 380, indicating non-turbulent flow. Smaller vessels with lower flow rates would be less likely to show turbulence, suggesting no significant error.

Only five data points are available to define the blue line in Figure 3. This is because we required a simultaneously acquired catheter manometry study of the sagittal sinus and a pressure transducer study of the ICP. Studies where the ICP measurement was performed hours to weeks apart from the venous study were rejected due to the expected pressure drift which would occur over time. In other available studies in the literature, a fluid manometer was used to measure the CSF pressure, which systematically lowers the pressure measurement. Iyer et al. performed LP immediately after the venography in their study but used a manometer, allowing several mL of CSF to be removed from the subarachnoid space to fill the manometer before the pressure measurement was taken [69]. Their results showed an ICP which was less than the venous pressure. This gave a non-interpretable negative pressure gradient across the arachnoid granulations.

One may suggest that the fifth data point, i.e., from Tariq et al. [10] is a long way removed from the other data points and may be an outlier. Tariq et al. [10] did not have a formal control group; ten of their patients had pituitary adenomas (adenomas would not be expected to alter the CSF formation rate and may be a surrogate control group). The adenoma group had a rate measured at 0.48 mL/min [10], which is similar to Qvarlander et al.’s reference rate of 0.4 mL/min [22], suggesting no obvious source for a systematic error in Tariq et al.’s [10] study. Our model provides an explanation for this rather high fluid flow rate. Figure 2 indicates that there is a very high net pressure gradient across the capillaries, which could drive this flow.

We have assumed the R_out_ to be normal at 10 mmHg/mL/min. Lalou et al. measured the R_out_ in their cohort to be 5.2 mmHg/mL/min, with this figure taking into account the increase in venous pressure which occurred whilst the CSF infusion was undertaken. They did not, however, take into account the change in the outflow resistance which would have occurred during the infusion. As already discussed, Welch and Friedman found that the critical opening pressure for the arachnoid granulations is between 1.5 and 3.7 mmHg [29]. At the beginning of their study, Lalou et al.’s [6] cohort had a pressure gradient across the granulations of 1.8 mmHg, meaning almost all of the valves were closed and therefore most of the CSF absorption was occurring across an accessory pathway (we will denote the resistance of this pathway R_1_). After the infusion, the pressure gradient across the granulations was increased to 4.9 mmHg and therefore the granulations would all be fully open. Their resistance would have reduced to 10 mmHg/mL/min (as is normal for fully open granulations), we will denote this resistance R_2_. Now, when there are two resistors operating in parallel, the formula for finding the total resistance is:

(15) 1R1+1R2=1Rtot

If *R*_2_ is 10 mmHg/mL/min and *R_tot_* is 5.2 mmHg (as Lalou et al. [6] found), then *R*_1_ (the resistance of the collateral pathways) is 10.8 mmHg, i.e., close to the figure we used for our modelling. Therefore, patients with IIH and without CSF pressure manipulation have a total outflow resistance of close to 10.8 mmHg/min. This figure would be somewhat lower in Liu et al.’s [5] cohort because the pressure gradient across the granulations would be 2.7 mmHg and some of the granulations would therefore be draining fluid. Therefore, we believe our compromise figure for R_out_ of 10 mmHg/mL/min is justified. We have performed a sensitivity analysis to look at the outcomes if the total the R_out_ was different to that which we used. If the R_out_ were doubled, then the calculated capillary TMP would not change because this part of the model does not use this parameter. However, the hydraulic conductivity coefficient for the capillaries would decrease to 0.06 mL/min/mmHg and the reflection coefficient would increase to 0.66. If we halved the R_out_ to 5 mmHg/mL/min, then the hydraulic conductivity coefficient would increase to 0.24 mL/min/mmHg and the reflection coefficient reduce to 0.46. Neither of these alternatives materially affect the outcome of this study.

We have assumed the mean arterial inflow pressure to be normal in IIH at 100 mmHg. Systemic hypertension is listed as a comorbidity of IIH in 20% of patients in one study [70] and 32% in another [71]. This would suggest that the majority of individuals (68–80%) with IIH are normotensive. However, even in those with a diagnosis of systemic hypertension, we expect many of those should have been successfully treated and thus have normal arterial pressures. Therefore, we used the normal figure in our modelling. We decided to perform a sensitivity analysis by increasing the arterial inflow pressure to 130 mmHg (being less than the cutoff for autoregulation failure at 150 mmHg) [23]. This analysis found that the arterial pressure does not alter the capillary TMP because the arterial resistance must increase to keep the blood flow from increasing. A 30% increase in arterial inflow pressure required a 56.6% increase in arterial resistance. The increased pressure drop across the arteries buffered the capillaries and, therefore, means that the capillary pressures remain unchanged. Similarly, it is suggested that an increase in ICP will reduce the CBF, but this only occurs when the limit of autoregulation is reached. This occurs at a pressure of 40 mmHg [72]. Our modelling is always below this figure. In the modelling of the CSF drainage (Figure 1F), we estimated a sinus pressure of 15.4 mmHg because this was the final sinus pressure found in the largest cohort study into IIH, where the sinus stenoses were reversed by stenting [28]. Review of the study by Lalou et al. [6] indicates that their sinus pressures stabilised at 16 mmHg once the CSF pressure in their cohort was reduced to below the sinus pressure (i.e., fully dilating the reversible stenoses). This is not materially different to the figure we used. This would suggest that we are correct in assuming that the residual sinus pressure is about 15–16 mmHg once the reversible sinus collapse is reversed with drainage. We cannot exclude the possibility that some patients may have irreversible stenosis in their sinuses (the number seems low given the Ahmed et al. study which fully dilated their sinuses and given that Lalou et al. give a similar figure). A sensitivity analysis was undertaken to look at the possibility of higher sinus pressures following drainage. This indicated that an increase in the venous sinus pressure of 20% will only lead to an 8% change in capillary TMP. This is because the model required a reduction in arterial and capillary resistance to maintain the blood flow to overcome the higher venous pressures. Thus, we believe our estimate for sinus pressure in drainage is reasonable.

The model sets the capillary volume to be unchanged as the capillary TMP is reduced below normal. This is because the critical buckling pressure for such a small tube as a capillary is very high and is never approached in the model [73]. We set the upper volume of the capillaries to be increased by 44% at a capillary TMP of 37.9 mmHg. Capillaries, being viscoelastic tubes, will have a size depending on the transmural pressure, wall thickness, resting diameter and the elastic modulus. The upper bound will be obtained when the elastic limit is reached. These data are not available for humans but in rats, the volume of the capillaries does not decrease below the normal capillary flow rate and increases by 44% when maximal a blood flow rate is achieved (i.e., the arteries are fully dilated) [24]. In our original study [11], we set the increase in size of the capillaries to 44% when we modelled the upper level of ICP autoregulation at 40 mmHg because, by definition, if the limit of autoregulation was reached, then the arterioles would be maximally dilated, so the capillary pressure should be maximal at this point. Figure 1D from this paper shows the average capillary TMP to be 37.9 at this point. Despite the assumptions involved, the predicted total blood volume was increased by 66%. In a primate model, Grubb increased the ICP from 8.6 to 71 mmHg, which increased the CBV by 66% [74] (identical to our findings); increasing the ICP further to 94 mmHg did not alter the CBV but did reduce the CBF. This indicated the autoregulation limit was transgressed at 94 mmHg and the elastic limit for the venous distension was also probably reached. These findings are identical to our findings that raising the ICP to the limit of autoregulation increases the CBV by 66%. Thus, we would contend that our modelling is accurate enough for our current purposes.

Some of the data we required are not available from human studies. In their absence, animal studies were utilised. This is exemplified by the data linking dilatation of the capillaries to the changes in TMP, which were taken from rat studies. The brain capillary of a rat averages 7.4 µm, with a wall thickness of 0.5 µm [75]. In humans, the brain capillaries vary from 7.0 to 9.0 µm, with a wall thickness of 0.4–0.75 µm [76]. They are not significantly different. Given the similarity in size and function of rat to human capillaries, we suggest that our expectation they have similar distension properties is reasonable. The normal venous TMP was obtained from primate studies. Given that primates and humans are close phylogenetically, we assume that this metric in primates is similar to that in humans. Ultimately, we have no way of knowing if the animal data closely approximate human findings, so this is a limitation.

### 4.5. Opportunities for Further Study

The model predicts that the CSF_fr_ varies as a function of the capillary TMP. The capillary pressure could be directly measured using an arterial wedge pressure technique in an animal model where IIH is induced by limiting the venous outflow. This would be too invasive to attempt in humans.

Our model suggests that the opening of the BBB is behind the over production of CSF when the ICP is reduced to zero. When an IIH patient is treated by using a ventriculoperitoneal shunt, the ICP is often set to 10 cmH2O or 7.4 mmHg. The model would predict that the flow through the shunt should be elevated if the patient continued to have elevated venous pressure (i.e., were morbidly obese) but may actually reduce to below normal if the transverse sinuses were no longer compressed and normal arachnoid granulation absorption resumed. Newer devises are available to measure CSF flow in shunt tubes in vivo to test this suggestion.

Further research could correlate the CSF formation rate in an experimental animal model of IIH with the use of tetracycline and retinoic acid drugs to try to confirm the suggestion that they alter the CSF formation rate.

The arachnoid granulations are smaller in IIH than normal, suggesting that they do not operate to absorb fluid. If, as we suggest, they begin to operate during an infusion study, then, to confirm this, their size could be measured with MRI during the infusion to see if they enlarge.

## 5. Conclusions

A reduction in the pressure gradient between the subarachnoid space and the venous sinuses suggests that the elevated ICP in IIH is due to an elevation in the venous pressure. Our modelling indicates that the CSF formation rate varies with the capillary transmural pressure and suggests that an opening of the BBB allows increased absorption or excretion of CSF via the accessory pathway provided by the capillary bed. Our modelling suggests that drugs which stabilise the BBB may promote IIH by blocking this accessory pathway to absorption. Anaemia may promote IIH by increasing the capillary transmural pressure and CSF formation rate as well as increasing the venous outflow pressure.

## Figures and Tables

**Table 1:** Results of modelling.

Study	ICP mmHg	SSS Pressure mmHg	CSF Formation Rate mL/min	Capillary TMP mmHg
Cagnazzo et al. [7]	22.0	20.9	0.11	10.8
Lalou et al. [6] Baseline	27.0	25.2	0.18	11.5
Liu [5] et al.	42.2	39.5	0.27	12.1
Lalou et al. [6] Infusion	38.0	33.1	0.49	13.9
Tariq et al. [10]	0.0	15.4	1.43	22.0

## Data Availability

All data are contained within the article.
